# The impact of professional learning communities on pre-service teachers’ professional commitment

**DOI:** 10.3389/fpsyg.2023.1153016

**Published:** 2023-06-28

**Authors:** Chunsong Cheng, Jinzhen Zhao

**Affiliations:** ^1^College of Teacher Education, Zhejiang Normal University, Zhejiang Province, China; ^2^College of Foreign Languages, Quzhou University, Zhejiang Province, China

**Keywords:** professional learning community, workshops for teaching competitions, pre-service teacher, impact, professional commitment

## Abstract

While in-service teachers’ professional commitment has gained significant attention in recent years, researches on pre-service teachers’ professional commitment is still insufficient, particularly with regard to the effect that professional learning communities play on pre-service teachers’ professional commitment. In this context, this study employed mixed methods to investigate the impact of workshops for teaching competitions as a professional learning community on pre-service teachers’ professional commitment in China. A questionnaire survey was administered to pre-service teachers who had workshop experiences (*n* = 43) and their classmates who did not have workshop experiences (*n* = 98) to examine the effect of workshops for teaching competitions as a professional learning community on pre-service teachers’ professional commitment. Follow-up qualitative interviews with 5 pre-service teachers with workshop experiences were conducted to explain the reasons behind such effects. The results showed that workshop experiences had significant and positive effects on pre-service teachers’ professional commitment and the characteristics of shared vision, collaboration, and reflective dialogue affected their professional commitment from three perspectives: commitment to teaching as a career, personal time investment, and interest in professional development.

## 1. Introduction

The concept of professional learning communities (PLCs) has been one of the most widely discussed research topics in the last 30 years (Zhang et al., 2023). Numerous studies have suggested that well-implemented PLCs can significantly contribute to student learning outcomes (Louis and Marks, 1998; Lomos et al., 2011; Akiba and Liang, 2016), teacher professional development (McLaughlin and Talbert, 2001; Hipp and Huffman, 2010), and school reform (Stoll et al., 2006; Harris and Jones, 2010; Doğan and Adams, 2018). As a result, systems and schools are investing considerable efforts in developing themselves as professional learning communities. Undoubtedly, PLCs are becoming more and more widespread in the world and therefore are in the ascendant in educational practice and policy at this time (Hargreaves, 2007).

In China, PLCs have been around in the form of Teaching Research Groups (TRGs, jiaoyanzu) from school level to provincial level for decades since the founding of the People’s Republic of China when TRGs were established officially and formally to improve teaching quality (Ministry of Education, 1952a,b). Chinese students’ top performance in the international assessment programs such as TIMSS and PISA has attracted international attention (Zheng et al., 2019). The multilevel and multifaceted PLCs have been considered as a major contributor to students’ excellent performance (Cheng, 2011). Teachers participate in the activities, such as collective lesson planning, teaching competitions, peer class observation, and delivering open lessons, etc. organized by PLCs of different forms, especially by school-level TRGs which are composed of teachers teaching the same subject in the same school. Even in some resource-strained and remote rural areas, teachers participate in these professional learning activities (Zheng et al., 2016). Therefore, a growing number of studies have explored the effects of PLCs on teachers’ professional development, such as teacher efficacy (Zonoubi et al., 2016; Zhang et al., 2023), job satisfaction (Zhang and Yuan, 2020; Zhang et al., 2023), and teacher commitment to students (Lee et al., 2011; Zheng et al., 2021).

In addition to the PLCs for in-service teachers, there are PLCs for pre-service teachers in the form of workshops for teaching competitions in many teacher-education-oriented colleges or universities. They provide opportunities for pre-service teachers’ professional development, help pre-service teachers get more qualified for their future teaching, and also contribute much to the success of education in China. However, little research has examined the effects of workshops for teaching competitions on pre-service teachers’ professional development.

There are usually workshops for teaching competitions of different subjects such as Chinese, English, Mathematics, and Science etc. in the teacher-education-oriented colleges or universities. In view of the research gaps mentioned above, the current study attempts to conduct a case study on workshops for English teaching competitions (WETCs) to explore the effect of workshops for teaching competitions on pre-service teachers’ professional commitment in China. It aims to explore whether their participation in the workshops enhance their personal time investment in activities related to teaching, their interest in professional development and their commitment to teaching as a career. Specifically, two research questions will be addressed in the study.

1. Do WETCs as a professional learning community improve pre-service teachers’ professional commitment?
2. How do WETCs practices affect pre-service teachers’ professional commitment?

## 2. Literature review

### 2.1. Teachers’ professional commitment

Commitment is individuals’ psychological bond or identification with an object that represents a special meaning or importance (Mowday et al., 1982; Buchanan, 1997). A committed employee is more likely to desire to be affiliated with the object, believe in the values and goals of the object, and exert effort beyond minimal expectations for the object (Firestone and Pennell, 1993).

In teacher education, teacher commitment is defined as teachers’ psychological attachment to professional institutions, to the teaching profession, and to colleagues, students and their parents (Park, 2005; Lee et al., 2011). This means teacher commitment is multifaceted and it is associated with various commitment objects such as students, the teaching profession, and school organizations. In other words, there are three types of teacher commitment, i.e., teacher commitment to the teaching profession (professional commitment), to the school (organizational commitment) and to students (student commitment).

Professional commitment is a psychological attachment or bond to the teaching profession (Firestone and Pennell, 1993). It implies the degree to which teachers value and feel connected to the teaching profession (Lamote and Engels, 2010) and the degree to which teachers are engaged in carrying out various specific tasks in the workplace (Brown and Leigh, 1996). Thus, teachers who are committed to their teaching profession are willing to exert considerable efforts for teaching and considered to be more satisfied with the work.

Previous studies show that professional commitment is the primary motivator of persistence and effort for pre-service learning to teach, thus having a great impact on their professional development. The stronger their professional commitment is, the more energized pre-service teachers feel to learn to teach (Durksen and Klassen, 2012). Parkes (1989) studied the impact of British pre-service teachers’ professional commitment on their performance in teacher education. It was found that professional commitment was positively related to performance. According to the study conducted by Fokkens-Bruinsma and Canrinus (2015) in the Netherlands, highly committed pre-service teachers are more likely to complete teacher education programs than their uncommitted counterparts.

What is more, previous studies indicate that professional commitment is one of key factors deciding whether pre-service teachers enter the teaching profession and influencing their intention in the teaching profession after graduation (Lam et al., 1995; Rots et al., 2007; Wang et al., 2021). The stronger the commitment is, the more likely pre-service teachers would plan to stay in the teaching profession (Lam et al., 1995). Rots et al. (2007, 2010) reported, highly committed pre-service teachers have clearer intentions to keep a teaching career goal but lower attrition from teaching. Their psychological attachment to the teaching profession rules out other professions as their primary career goals (Wang et al., 2021).

### 2.2. WETC as a PLC

In China, there are provincial and national teaching competitions for pre-service teachers. Even before they enter the provincial teaching competitions, they participate in the competitions held by the college or university. In both provincial and national teaching competitions, pre-service teachers are asked to design a lesson plan and conduct a micro-teaching according to the teaching material given to them. In order to help pre-service teachers have a good performance in the competitions, workshops for teaching competitions have been established in many four-year bachelor programs of education in the universities. The workshops are usually presided over and run by one or more supervisors whose major concern is improving pre-service teachers’ teaching of a subject in the primary or secondary school. They study the subject teaching or have some teaching experience in elementary or secondary schools.

Although there is no universal definition of a professional learning community, there is a consensus that one exists when a group of teachers share and critically interrogate their practice in an ongoing, reflective, collaborative, learning-oriented, growth promoting way (Toole and Louis, 2002). An underlying assumption is that the teachers involved see the group as a serious collective enterprise (King and Newmann, 2001). It is also generally agreed that effective professional learning communities have the capacity to promote and sustain the learning of professionals with the collective purpose of enhancing student learning (Louis et al., 1995).

In WETCs, pre-service EFL teachers under the supervision of supervisor(s) come together as a team to help each other in a reflective and collaborative way. WETCs are different from one-time workshops. WETC participants can build long-term connections with the supervisor and other pre-service teachers from other classes. Under the supervisor’s supervision, pre-service teachers collectively interrogate and improve their EFL teaching performance by means of mutual learning and collaborative reflection with the purpose of promoting their professionalism (shared vision on learning). Specifically, under the guidance of the supervisor, they collectively design lesson plans, deliver micro-teaching, observe master teachers’ classroom teaching, and conduct peer observation of micro-teaching (deprivatized practice). In addition, they discuss the major or difficult issues in their lesson plans and micro-teaching (collaborative learning), and give comment on each other’s lesson plans and micro-teaching (reflective dialogue). The supervisor also makes comments and suggestions on their works. They learn not only from peers but experts as well in learning communities. According to the activities and organization above-mentioned, it is found that WETCs have the core characteristics of PLCs, such as shared vision on learning, collaborative learning, deprivatized practice and reflective dialogue (Stoll et al., 2006; Vescio et al., 2008; Vangrieken et al., 2017). In this way it is considered as a typical PLC for pre-service teachers.

Previous studies indicate that PLCs for in-service teachers hold promise for improving teacher development (Zhang and Yuan, 2020). Participants of PLCs report a higher level of professional development, including enhanced collective responsibility for student learning (Louis et al., 1995), improved teaching effectiveness (Vescio et al., 2008), an increased organizational commitment (Hausman and Goldring, 2001), and a reduced sense of isolation (Dalgarno and Colgan, 2007). However, there is no empirical evidence as to whether and how PLCs for pre-service teachers can improve their professional development. Since professional commitment is considered as the primary motivator of persistence and effort for pre-service learning to teach and has a great impact on their professional development (Wang et al., 2021), the current study attempts to conduct a case study of WETCs in China and explore whether and how PLCs for pre-service teachers can improve their professional commitment.

## 3. Methods

Mix methods were adopted in the study. The first question was addressed through a quantitative study, in which a questionnaire survey was conducted to examine the effect of WETCs on pre-service EFL teachers’ professional commitment. The second question was answered by an explanatory qualitative research, in which semi-structured interviews were conducted after the questionnaire survey to explain how the effects identified from the questionnaire survey occurred as perceived by the pre-service teachers.

### 3.1. Research respondents

In China, initial teacher education is university-based. In all the undergraduate teacher education programs, both subject courses and teacher education courses are offered. For example, in the four-year Bachelor programs of EFL teacher education, besides the compulsory subject courses such as English reading, English writing, English speaking, English listening, linguistics and English literature, teacher education courses such as EFL pedagogy, EFL lesson planning, EFL micro-teaching, and teaching practicum are also usually compulsory. The difference among the curricula in different universities lies in the number of credits of some courses or the semester in which the courses are provided.

In the study, altogether 141 respondents (aged from 21 to 23 years old) were included. All of them were from the WETCs in Zhejiang Province. The particular WETCs in Zhejiang Province were selected for two reasons. First, the teacher-education-oriented universities in Zhejiang Province have longer history and more experience of workshops for pre-service teachers’ teaching competitions as the provincial teaching competitions for pre-service teachers in China started in Zhejiang Province, and even the national teaching competitions for pre-service teachers are a copy of Zhejiang provincial competitions and held by Zhejiang Normal University. Second, the authors were familiar with the supervisors of the WETCs. With the help of the supervisors, respondents were free to speak in the interview.

There are two semesters in an academic year in China. The WETCs are offered to students when they are in the sixth and seventh semesters. The provincial and national teaching competitions are usually held at the end of each year. In the study, all of the respondents were in the last year of their undergraduate study in the teacher education program. Among them, 43 respondents who attended the WETC were in the experimental group and agreed to complete the questionnaire survey on a voluntary base. The rest 98 respondents who did not have the WETC experience were included in the control group and agreed to complete the questionnaire survey on a voluntary base. Since most students in EFL teacher Education programs are female, among the 43 respondents in the experiment group, 37 were female and only 6 were male. Among the 98 respondents in the control group, 81 were female and only 17 were male.

### 3.2. Research procedure

The WETCs usually went from students’ sixth semester through the end of their seventh semester. After the WETCs ended, with the help of WETC supervisors, the authors asked the WETC participants to be the respondents in the experimental group and participate in the questionnaire survey on a voluntary basis. Their classmates who did not participated in the workshop were invited to be respondents in the control group. Finally, 43 respondents in the experimental group and 98 respondents in the control group completed the questionnaire survey. The questionnaire was anonymous and all the respondents were informed that the data would only be used for research purpose.

After the questionnaire survey, semi-structured interviews were conducted with 5 participants in the experimental group. The 5 participants were selected to be interviewed because they gained much professional growth from the WETC experience, such as winning teaching prizes, being offered jobs in reputational schools.

### 3.3. Research instruments

Pre-service teachers’ professional commitment was measured using the questionnaire developed by Lauermann et al. (2017). The questionnaire included three subscales: commitment to teaching as a career, interest in professional development, and personal time investment. The questionnaire was translated by the first author and back translated by the second author.

Commitment to teaching as a career was assessed with two items from the FIT-Choice framework (Watt and Richardson, 2007) that capture satisfaction with one’s career choice and planned persistence in teaching (*α* = 0.896. How satisfied are you with your choice of becoming an EFL teacher? How sure are you that you want to work as an EFL teacher in the future?). The respondents rated each item on a seven-point Likert scale ranging from 1 (Not at all) to 7 (Very much).

Interest in professional development was assessed with four questions (*α* = 0.806): How important would it be/ is it to you to participate in professional development activities that… (1) focus on pedagogical knowledge of English teaching? (2) focus on classroom management skills? (3) focus on alternative teaching practices? (4) focus on subject specific knowledge of English? The participants responded on a seven-point Likert scale ranging from 1 (Not at all) to 7 (Very much).

Personal time investment measured the amount of personal time the participants were willing to invest in professional activities and tasks. It was assessed with five questions (*α* = 0.922): How much of your personal time are you willing to invest … (1) to work with students? (2) to communicate with parents? (3) to help students? (4) to prepare good lessons? (5) to improve your teaching? The participants responded on a seven-point Likert scale ranging from 1 (None) to 7 (Most of it).

This validated 11-item scale showed acceptable reliability (Cronbach’s alpha coefficients of global professional commitment to sub-dimensions ranged from 0.806 to 0.970) and good structural validity (see Lauermann et al., 2017).

The second research question was addressed by means of a follow-up qualitative research, in which semi-structured interviews were conducted after the respondents finished the questionnaire survey. All interviews were conducted in Chinese and each lasted approximately 40–60 min. During the interviews, the participants were asked to share their individual experiences with WETC practices and describe how these experiences affected their commitment toward EFL teaching. All of the interviews were audio-recorded and transcribed by the first author and checked by the second author. The Chinese data were first analyzed and then translated into English by both authors.

### 3.4. Data analysis plan

The quantitative data were analyzed using the statistical package SPSS version 27. First descriptive statistics such as mean scores of each question and each subscale were calculated. Then, to answer the first research question, the difference was tested between the experimental group and the control group in terms of the three subscales of professional commitment. Assumptions were checked prior to conducting relevant tests in order to ensure appropriate tests (i.e., parametric or non-parametric) were used.

**Figure 1:**
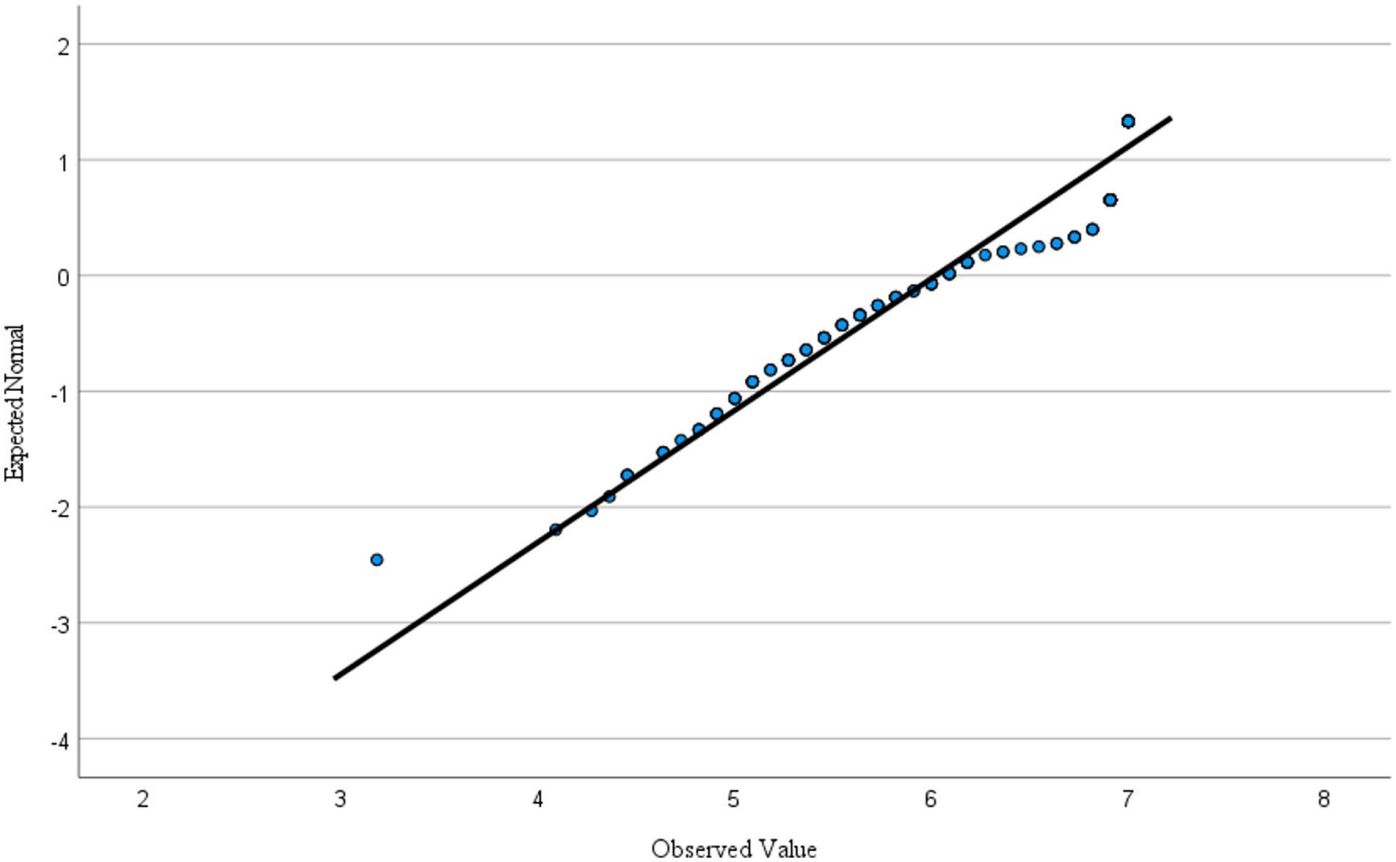
Normal Q-Q Plot of mean score of professional commitment.

The result of normality test with Kolmogorov– Smirnov significance value (<0.001) indicated a violation of the assumption of normality. However, such a result is quite common because of small sample size or unbalanced samples, and the actual shape of the distribution of scores indicated a reasonable normal distribution. This outcome was supported by an inspection of the normal probability plots (Normal Q–Q plot) that suggested a normal distribution (See Figure 1). The Levene test result indicated a significance value (<0.001) suggesting a violation of the assumption of homogeneity of variance, leading to the use of the non-parametric Mann–Whitney *U* tests to test whether the two groups differ significantly in professional commitment.

All of the interviews were audio-recorded and transcribed by the first author. The Chinese data were first analyzed and then translated into English. The data were analyzed in the following ways: “(1) making sense of the narratives, (2) coding for themes, (3) reconstructing the narratives for a storyline, and (4) telling and retelling, living and reliving the stories” (Liu and Xu, 2011: 591). Narrative excerpts are included in the section of Findings and Discussions, where participants are given pseudonyms to maintain anonymity. For the qualitative part, the authors transcribed and read repeatedly the data. Transcripts were also sent back to the participants for correction and approval. They were told that they could edit the transcripts for accuracy and clarity.

## 4. Findings

### 4.1. Do WETC experiences improve pre-service teachers’ professional commitment?

Many studies (e.g., Vescio et al., 2008; Doğan and Adams, 2018) have revealed that PLCs are a beneficial structural condition for promoting teacher development. This study showed that PLCs benefited pre-service teachers more than that. WETC experiences motivated them to be more committed to their future teaching profession. Mann–Whitney *U* tests showed the null hypotheses were rejected and pre-service EFL teachers who had WETC experiences were significantly more committed than their counterparts who had no such experiences. Table 1 is a summary of descriptive statistics and the results of Mann–Whitney *U* tests.

**Table 1:** Comparison of experimental group and control group on professional commitment.

Subscale	Experimental group (*n* = 43)		Control group (*n* = 98)		*U*	*Z*	Sig.
	*M*	*SD*	*M*	*SD*			
CTA	6.76	0.35	5.03	1.31	442.00	−7.59	0.000
IPD	6.94	0.12	5.66	0.90	357.00	−8.01	0.000
PTI	6.88	0.15	5.87	0.64	467.50	−7.49	0.000

CTA, commitment to teaching; IPD, interest in professional development; PTI, personal time investment.

As depicted in Table 1, the mean score of the experimental group’s commitment to teaching, interest in professional development and personal time investment were, respectively, 6.76, 6.94 and 6.88. These indicated that pre-service EFL teachers who had WETC experiences were extremely interested in professional development, extremely willing to invest their personal time on teaching activities, and extremely likely to take EFL teaching as career. The mean score of the control group’s commitment to teaching, interest in professional development and personal time investment were, respectively, 5.03, 5.66 and 5.87. The experimental group’s mean scores were significantly higher than those of the control group. This means that comparatively speaking, pre-service EFL teachers with WETC experiences were more committed than those without WETC experiences. Inspections of the mean differences in the three subscales indicated that the biggest difference between these two groups of pre-service EFL teachers on professional commitment was the difference in commitment to teaching as a career (MD = 1.73). The mean score of control group’s commitment to teaching was only 5.03, but that of the experimental group was as high as 6.76.

Further inspections of the mean scores of each question also showed that the difference of the three subscales between these two groups of pre-service EFL teachers varied from question to question. Table 2 shows the difference between the experimental group and the control group in terms of each question in the three subscales.

**Table 2:** Comparison of experimental group and control group on each question.

Subscale	Questions	Experimental group (*n* = 43)		Control group (*n* = 98)		*U*	*Z*	*Sig.*
		*M*	*SD*	*M*	*SD*			
CTA	Question 1	6.70	0.46	5.10	1.33	576.00	−7.08	0.000
	Question 2	6.84	0.37	4.97	1.35	405.50	−7.88	0.000
IPD	Question 1	7.00	0.00	5.75	1.04	580.50	−7.38	0.000
	Question 2	6.80	0.41	5.46	0.95	520.50	−7.44	0.000
	Question 3	6.98	0.15	5.54	1.00	432.50	−7.95	0.000
	Question 4	7.00	0.00	5.91	1.00	645.00	−7.15	0.000
PTI	Question 1	6.95	0.21	5.71	0.77	381.50	−8.20	0.000
	Question 2	6.70	0.51	5.65	0.83	686.50	−6.72	0.000
	Question 3	6.95	0.21	5.72	0.76	381.50	−8.21	0.000
	Question 4	6.91	0.29	6.13	0.59	651.00	−7.33	0.000
	Question 5	6.90	0.29	6.14	0.54	653.00	−7.35	0.000

As shown in Table 2, the mean score of each question in the experimental group ranges from 6.70 to 7.00, which means they were extremely committed in all perspectives. The mean score of each question in the control group ranges from 4.97 to 6.14. The highest score (6.14) is from Question 5 of PTI, the lowest (4.97) from Question 2 of CTA. Furthermore, Mann–Whitney *U* tests showed that there was significant difference among the mean scores of some questions of IPD and PTI in the control group. For example, in the subscale of PTI, the mean scores of Question 4 (6.13) and Question 5 (6.14) were higher than those of Question 1, Question 2, and Question 3. This means that pre-service EFL teachers without WECT experiences were more willing to prepare good lessons and to improve their teaching than to work with students, to communicate with parents and to help students.

### 4.2. How do WETC experiences affect pre-service teachers’ professional commitment?

In order to discover how WETC experiences affect pre-service teachers’ professional commitment, an interview study was conducted with 5 respondents who had WETC experiences. By analyzing the interview transcript, we found that three characteristics of PLCs were significant predictors of EFL pre-service teachers’ professional commitment.

The first significant predictor is shared vision. WETC participants worked with a group of people who had the same vision and goals. All of them participated in WETCs in order to improve their professional competence. The shared vision helped create throughout the workshop a progressive learning culture which affected their professional commitment positively by encouraging and stimulating the participants to attend the workshop activities and make more efforts in their professional development. As the interviewee Ge reported,

As the saying goes “If you want to go fast, go alone; if you want to go far, go together,” our teaching professional development is the case. Before I participated in the WETC, I learned the way of practicing my lesson planning from the course of Lesson Planning, but I could not persist on practicing lesson plans alone. In the WETC, I was always stimulated and encouraged by other members’ persistence and diligence. Sometimes, I was even pushed. Gradually I got used to the practice and got to enjoy these activities beneficial to my professional development. Now I really miss the days when we worked together.

The preceding quote indicated that the company of peers who had shared vision played an important role in pre-service teachers’ leaning and their professional commitment was affected positively by the progressive learning culture. The culture facilitated pre-service teachers’ continuous learning. This opinion was shared by all the interviewees. Besides, their professional commitment was also affected by the WETC participants of the previous years. Each year there were WETC participants who won the national or provincial English teaching prizes or received job offers from some prestigious schools. They were often invited to introduce their WETC experience to the new participants of WETCs. They set good examples for the new participants. For example, pre-service teachers interviewed reported that they always considered some of the participants of the previous year as their role models.

Before I participated in the workshop, I was always told that the job market for pre-service EFL teachers was very competitive because the vacancies for EFL teachers were much less than those for Chinese or Math teachers in Zhejiang Province. I was afraid that I would not be employed by any school, but my WETC experiences told me those who had good professional competency could still find teaching positions. Many of those who participated in the WETC in the previous years are working in very famous elementary or middle schools now, so I’m confident I will receive job offers from middle schools. Before I participated in the workshop, I hoped that I could teach at an elementary school, but now I think I’ll be qualified to teach middle school students (Ge).

I always take Mo as my role model. He was in the WETC last year and now he is teaching at a very reputational middle school in Hangzhou. He said the WETC experience which improved his professional competence greatly helped him get recruited to such a prestigious school. He often expressed his great gratitude to the WETC. I will participate in the WETC activities like him to improve my professional competence. I’m very confident that I will be a qualified English teacher and successfully receive job offers when I graduate (Fang).

The two quotes showed that new WETC participants were encouraged and motivated by the previous participants. After WETC learning they were more confident about their teaching profession in the future than before. They could see good job opportunities brought about by WETC experiences. In this study, the WETC participants believed that they would successfully receive job offers when they graduated. As a reward, their confidence influenced how satisfactory they were with their choice of becoming/being an EFL teacher and how sure they were to want to work as an EFL teacher in the future. In this way, their confidence made them more committed.

The second significant predictor is collaboration. WETCs set clear goals for improving participants’ professional competence by engaging them into many activities. As WETC participants took part in collaborative activities, such as lesson study, collective lesson planning, and micro-teaching, they collaborated with each other and improved their professional competence. As the saying goes “Great things may be done by mass effort,” they improved their teacher competency by collaboration. Gradually they learned to set more appropriate teaching objectives, design better and more diverse classroom activities, implement more proper evaluation activities. Such improvement helped them develop a sense of achievement and satisfaction which as a result affected their commitment to teaching as a career and encouraged them to spend more efforts in these activities. They believed they could be a good English teacher if only they kept attending such activities and they knew the feasible ways of improving their professional competence.

Many collaborative activities were offered in WETCs, such as peer observation, collective lesson planning, observing expert teachers’ teaching. The more activities I participated in, the more progress I made. I can set specific teaching objectives and design better lesson plans now. I’m willing to spend more time learning to be a good English teacher (Lin).

The preceding quote from the interview indicated that the progress she made affected her willingness to invest in professional activities and tasks. She experienced the proper ways of improving her professional competence, so she became confident in her future English teaching career. On the other hand, the progress stimulated her motivation to be a better English teacher applicant. She not only interested in the activities that are beneficial to improving her professional development, but also willing to invest time to these activities. She believed she could be a good English teacher if only they kept attending such activities even after she finished the WETC learning.

Pre-service teachers have various motivations to enter teaching profession. Some are motivated to impact students (altruism), some are motivated by an innate calling to the profession (intrinsic motivation), and some are motivated from observing and interacting with others (socialization influences) (Kwok et al., 2022). The study found that pre-service EFL teachers were motivated differently to enter the four-year Bachelor Program of English Education. Among the five interviewees, 3 of them reported that they had intrinsic motivation to teach English. They chose the program mostly because they loved English teaching. One reported she was motivated mostly by the social status of teaching profession and one reported she wanted to be a translator. As the interviewee Li reported,

I planned to study in the Bachelor Program of English Education with the purpose of improving my English so that I could do business translation or literature translation when I graduate, but WETC activities such as collective lesson planning, peer observation of micro-teaching changed my plan. I found these activities so interesting and they made me a qualified EFL teacher. I like these professional development activities (Li).

In Li’s case, she planned to be a translator after graduation, but WETC changed her mind because WETC activities built her interest in EFL teaching. According to the quantitative analysis, among the three subscales, the biggest difference between these two groups of pre-service teachers’ professional commitment lies in their commitment to teaching as a career. Those with WECT experiences were far more confident that they would be able to be EFL teachers when they graduated. According to the qualitative analysis, their confidence had great impact on their commitment to teaching as a career and could explain the reason for the biggest difference. In China, teachers at all levels of the educational system possess considerable social status and are highly respected (Zhang et al., 2018). According to the 2013 Global Teacher Status Index, teachers in China rank the first among the 21 surveyed countries such as the UK, the USA, France (Dolton and Marcenaro-Gutierrez, 2013). This Index also shows that two-thirds of the 21 countries surveyed see the status of their teachers as being most similar to social work (i.e., lowly ranked compared to other professions), but in China teachers are ranked as being on the same level as the medical practitioners. The study discovered that those with WETC experiences were more likely to be motivated by the intrinsic motivation even if they were motivated by teachers’ social status when they entered the teacher education program. They gained confidence from their community learning and experienced the feasible way of promoting their professional competence. Their confidence in turn motivated them to be more interested in professional development and encouraged them to invest more time on their professional competence.

The third significant predictor is reflective dialogue which bridges theories and practice. Reflective dialogue is a conversation wherein two or more colleagues reflect with each other on serious educational issues or problems related to instructional practice and specific students’ learning (Louis and Marks, 1998; Stoll et al., 2006; Brown et al., 2017). Under the supervisor’s guidance, the participants reflected on the teaching objectives they set, the instructional activities they designed, the questions they asked, and the assignments they designed. They discussed how they could make the teaching objectives more clearly stated and more specific, how they could make the instructional activities helpful to achieve teaching objectives and assignments more beneficial to improving students’ English proficiency and critical thinking, and how they could make the questions more easily understood by students. In this way, participants realized the importance of theories and teaching principles underlying the lesson plans and micro-teaching, found it interesting to make lesson plans and micro-teaching better and gained a better understanding of English teaching from different perspectives. They learned to connect theory and practice more effectively. As the interviewee Zhang reported,

My favorite activity is reflective dialogue after lesson planning and micro-teaching. The supervisor guided us to reflect on everything related to lesson plans and micro-teaching. Each time we were asked to reflect on the problems in teaching objectives we set, in instructional activities and assignments, even in the way questions were presented. The most important was that we could found solutions to the problems and understood the pedagogical theories and teaching principles underlying the solutions with the supervisor’s help. I was very happy with the process and was always looking forward to the activity (Zhang).

The quote showed that participant was very interested in reflective dialogues and always looked forward to the activities because these activities helped him gain a better understanding of English teaching. Reflective dialogues built them more committed in that he was more interested in and willing to participate in the professional activities. The study showed that reflective dialogue was a significant predictor of professional commitment. The result is similar to those of previous studies conducted to in-service teachers (Hausman and Goldring, 2001; Fransson and Frelin, 2016; Doğan and Adams, 2018), but surprisingly a little inconsistent with that of researches conducted by Zheng et al. (2021) who found in-service teachers in PLCs in China were likely to talk “the right nonsense” to maintain a harmonious atmosphere and interpersonal relationships, and this stopped them having critical and deep conversations. In this study, the pre-service teachers were less likely to talk “the right nonsense” in order to save each other’s face. They were guided by the supervisor to present their opinions from different perspectives to avoid superficial conversations. They were more likely to have critical and deep conversations and many constructional suggestions were raised each time in reflective dialogues.

## 5. Discussions

The quantitative findings showed that pre-service EFL teachers who had WETC experiences were more interested in professional development and more willing to invest personal time on teaching activities than their counterparts who had no WETC experiences. The biggest difference was those with WETC experiences were more likely to take EFL teaching as their career. Therefore, WETCs had significant and positive effects on pre-service EFL teachers’ professional commitment. Such findings are consistent with the results of previous research (e.g., Hausman and Goldring, 2001; Zheng et al., 2021) that PLCs help sustain and enhance teachers’ professional commitment.

**Figure 2:**
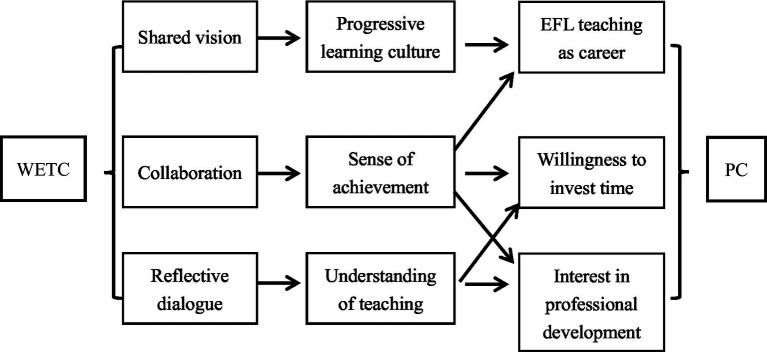
Mechanism of how WETCS influences pre-service teacher’ proffesional commitment.

The qualitative findings indicate that shared vision, collaborative efforts and reflective dialogue are the three characteristics of PLCs that influenced pre-service EFL teachers’ professional commitment significantly as Figure 2 shows. Shared vision helped create among the whole workshop a progressive learning culture. Collaboration helped develop among the participants a sense of achievement and satisfaction. Both the progressive learning culture and the sense of achievement and satisfaction helped improve their confidence to be an EFL teacher. In addition, the sense of achievement and satisfaction enhance their willingness to invest personal time on teaching and made them more interested in professional development activities. Reflective dialogues helped participants have a better understanding of EFL teaching and the connection between theory and practice, which make participants more interested in professional development activities and willing to spend more time on professional activities.

Both the quantitative and qualitative findings indicated that WETC experiences are beneficial to pre-service EFL teachers in many ways. The experiences improved their professional competence, helped them experience the feasible way for their future professional development, and enhanced their professional commitment. The most important is that pre-service teachers gained confidence from the experiences. They are far more confident than their classmates with no WETC experiences that they could be able to be EFL teachers when they graduated. Therefore, it was concluded that WETCs as professional learning communities are the way toward sustainable teacher professional development.

## 6. Conclusion and implications

The study explored the impact of WETCs as professional learning communities on pre-service EFL teachers’ professional commitment in China and how WETC experiences affect their professional commitment by means of a quantitative survey and follow-up qualitative interviews. Workshops as professional learning communities benefit pre-service teachers from different perspectives. The characteristics of PLCs: shared vision, collaborative efforts and reflective dialogue work together to develop pre-service teachers’ teaching competence and enhance and sustain their professional commitment. It may be concluded that WETCs as professional learning communities provide pre-service teachers with the way toward sustainable development for their future profession.

The study implied much for promoting pre-service teachers’ professional commitment. First, extra efforts are needed to cultivate committed teachers. Usually teacher education courses for pre-service EFL teachers such as Pedagogy, English language teaching methodology, studies of English curriculum and teaching materials, second language acquisition as well as teaching practicum are offered in undergraduate EFL teacher education programs of Chinese universities, but respondents became more confident after the WETC learning than before. They are more satisfied with their choice of becoming an EFL teacher and more sure that they want to work as an EFL teacher in the future. They are more confident that they would be qualified to be an EFL teacher when they graduated. According to the interview, WETCs played a very important role in enhancing the participants’ professional competence. Without the WETC, the participants could not persist on the professional development activities even though they learned the feasible way of improving their professional competence from the teacher education courses before they participated in the workshop. However, they successfully promoted their professional competence in the WETC. From the difference between these two groups of pre-service teachers’ commitment to teaching as a career, it can be referred that these teacher education courses alone are not enough to cultivate committed EFL teachers. Extra efforts or practices are needed for teacher candidates to be more qualified and more committed. Professional learning communities are good places in which they work collectively to improve their professional competence and enhance their professional commitment.

Second, pre-service teachers’ professional learning communities need supervisors. In the interview, the respondents mentioned the importance of supervisors. Thanks to the supervisors reflective dialogues were well organized. Supervisors have extensive experience in teaching and know much more about theories and educational policies. They know what theories pre-service teachers need to understand, what teaching methods they will apply in the future teaching profession, and what reflective skills they will employ in the reflection activities. On the one hand, it is very beneficial for pre-service teachers to learn directly from these supervisors’ professional competence, as this will greatly enhance pre-service teachers’ professional competence. On the other hand, supervisors can make plans for the community to work, to recommend right books for pre-service teachers to read, to organize reflective dialogues, and to give insightful comments and suggestions on their lesson plans and micro-teaching.

This study incudes limitations and directions for future research. First, this study is a cross-sectional study which compared two groups of pre-service EFL teachers’ professional commitment when they were in the last year of their undergraduate study. A longitudinal study may find more details of the impact of WETCs on their professional commitment and how their professional commitment get promoted when they are in the WETCs. Second, the quantitative data were based on respondents’ self-report. Future research should use multiple methods such as respondents’ journals, supervisors’ interview or observation, and their classmates’ interview or observation to investigate how WETCs impact pre-service EFL teachers’ professional commitment. Third, the study misses a social validity measure. If the social validity was assessed, the study would be more persuasive and more helpful to the teacher education programs in other countries.

## Data availability statement

The raw data supporting the conclusions of this article will be made available by the authors, without undue reservation.

## Ethics statement

The studies involving human participants were reviewed and approved by the Ethics Committee of the College of Teacher Education, Zhejiang Normal University. The patients/participants provided their written informed consent to participate in this study.

## Author contributions

CC: conceptualization, methodology, and writing – original draft preparation. CC and JZ: questionnaire, investigation, data analysis, writing – review and editing and supervision. All authors critically revised the manuscript and gave their final approval of the manuscript submitted for publication.

## Conflict of interest

The authors declare that the research was conducted in the absence of any commercial or financial relationships that could be construed as a potential conflict of interest.

## Publisher’s note

All claims expressed in this article are solely those of the authors and do not necessarily represent those of their affiliated organizations, or those of the publisher, the editors and the reviewers. Any product that may be evaluated in this article, or claim that may be made by its manufacturer, is not guaranteed or endorsed by the publisher.
